# Effect of Peroneal Electrical Stimulation and Ankle-Foot Orthosis on Gait Parameters and Ground Clearance Among Stroke Survivors

**DOI:** 10.7759/cureus.110867

**Published:** 2026-06-15

**Authors:** Janhavi Kulkarni, Suraj Kanase

**Affiliations:** 1 Department of Neurosciences, Krishna College of Physiotherapy, Krishna Vishwa Vidyapeeth (Deemed to Be University), Karad, IND

**Keywords:** functional independence, motor recovery, neurorehabilitation, stroke rehabilitation, therapeutic exercise

## Abstract

Background: Stroke often leads to foot drop and impaired gait due to weak dorsiflexors and poor motor control, resulting in reduced ground clearance and increased fall risk. Effective interventions are needed to improve gait safety and adaptability in poststroke rehabilitation.

Purpose: This study aimed to compare the effectiveness of functional electrical stimulation (FES) combined with an ankle-foot orthosis (AFO) vs. FES alone on gait parameters and ground clearance in stroke survivors.

Participants: Thirty stroke survivors with foot drop were randomly allocated into two groups: Group A (FES + AFO) and Group B (FES only). All participants were medically stable and able to walk at least 10 m independently or with minimal assistance.

Methods: Both groups underwent 40-minute intervention sessions, five days per week for three weeks, in addition to conventional gait training. FES was applied to the common peroneal nerve during the swing phase using a foot switch. Group A additionally used a lightweight posterior leaf-spring AFO during gait training and ambulation. Outcome measures included stride length, step length, cadence, ground clearance assessed using the Kinovea application, and dynamic balance assessed using the Dynamic Gait Index (DGI).

Results: Both groups demonstrated significant improvements in gait parameters following intervention. However, Group A (FES + AFO) showed greater improvement in DGI scores and ground clearance (mean ± standard deviation (SD)) compared with Group B (FES only) (mean ± SD), with statistically significant differences between groups (p < 0.05), indicating better gait adaptability and enhanced walking safety.

Conclusion: This study suggests that FES combined with AFO is more effective than FES alone in improving gait parameters and ground clearance in stroke survivors. Its novelty lies in the objective Kinovea-based assessment of ground clearance along with DGI. However, the findings are limited to short-term effects due to the three-week intervention period, and longer follow-up is needed to confirm sustained outcomes.

## Introduction

Stroke is a leading cause of long-term disability worldwide, leaving many survivors with motor deficits that severely impair walking ability [1]. Stroke is a prevalent cause of mortality and morbidity in most advanced countries. Stroke in India is associated with an annual incidence rate of between 90 and 220 cases per 100,000 population, representing 1,443,528 cases of ischemic stroke in a year [2]. Patients exhibit sustained motor impairments that limit their ability to perform activities of daily living independently. Most frequently, stroke leads to impairments in neurological functions, causing loss of sensory and movement function in one half of the body (hemiplegia) [3]. Other forms of impairment include inability to stand or walk, joint stiffness, reduced hand function, and communication difficulties. Motor recovery of the impaired limb requires regular exercise sessions, but this cannot be achieved effectively due to inadequate resources [4]. Despite the fact that almost 70% of patients with stroke recover their walking function, their ability to walk freely is hampered by spastic hemiparesis. Walking difficulties still significantly limit functional capacity and quality of life (QoL) of stroke survivors [5].

Approximately 20% of stroke patients develop foot drop as a sequela of spastic hemiparesis following stroke [6]. Foot drop is typically caused by weakness of the ankle dorsiflexors and spasticity of the plantar flexors, resulting in poor gait efficiency and increased risk of falls [7]. This condition may be associated with stereotypical paretic-limb gait abnormalities characterized by insufficiencies in hip and knee flexion during the swing phase of gait. Consequently, patients with poststroke foot drop are prone to dragging the paretic foot while walking. Typically, such a problem is partially addressed by hip abduction and pelvic tilt toward the affected side, but this approach is highly inefficient and ineffective. Moreover, excessive loading of the forepart of the foot and the lateral border during the stance phase of gait impairs the patient's ability to distribute load properly and maintain balance on the paretic limb. Overall, such a combination of disorders adversely affects locomotion capacity and the QoL of affected patients.

The standard mode of care in addressing these deficits is the custom-molded ankle-foot orthosis (AFO) and physiotherapy. An AFO, usually made of polypropylene or carbon fiber, is a brace worn on the lower leg to hold the foot and ankle in the correct position. The use of an AFO restricts the natural passive range of motion and flexibility of the ankle and foot, may limit walking ability on uneven terrain, and may be uncomfortable to use [8]. AFOs may reduce sensory feedback and impose mechanical restrictions during activities other than walking. Some patients also perceive them as cosmetically unappealing or inconvenient, which may reduce compliance or lead to rejection of the device.

The neuromuscular electrical stimulation approach makes use of the stimulation of nerves with electrical impulses to remedy the problem of weakness of muscle innervation that prevents joint movements in stroke survivors. This enables those with impaired limb mobility to regain control of muscle function and coordinate movement [9]. The use of electrical stimulation to treat movement problems dates back several decades, with studies on the treatment of foot drop using electrical nerve stimulation conducted since the sixties [10]. By causing the muscles to dorsiflex the ankle and subtalar joints, functional electrical stimulation (FES) enhances foot clearance and allows patients to more easily cope with uneven terrain compared with AFOs [11]. Additionally, studies have shown that stimulation of the common peroneal nerve can cause knee and hip flexion, helping with foot clearance during swing [12]. Due to the large population of patients suffering from stroke survivors, several research studies have been conducted over the past three to four decades on orthosis-based FES therapy. Externally induced dorsiflexion using FES was first introduced by Liberson et al. in 1961 as an alternative treatment for foot drop [10,13]. FES therapy has proven advantageous not only in its therapeutic aspects but also in its orthotic applications, due to improved walking velocity and reduced energy cost of walking [14]. Recovery of motor function following a stroke is highly dependent on the extent of initial impairment.

Therefore, the present study was designed to evaluate and compare the effects of FES, AFO, and their combined application on gait adaptability and ground clearance in stroke survivors. Although both interventions are widely used in clinical practice, limited evidence exists regarding their comparative effectiveness and the potential synergistic benefits of combining them. This study aimed to determine whether the combined use of FES and AFO provides superior improvements in gait parameters and minimum toe clearance than either intervention alone, thereby contributing to evidence-based clinical decision-making in poststroke gait rehabilitation.

## Materials and methods

Study design

This pre-post experimental study was conducted over a period of six months in 2025-2026 (starting from November 5, 2025, to May 5, 2026) at Krishna College of Physiotherapy, Karad, Maharashtra, which is equipped with facilities for gait training, FES, and orthotic fitting. Ethical clearance was obtained from the Institutional Ethics Committee of Krishna Vishwa Vidyapeeth, Deemed to be University (Ref. No.: KVV/IEC/10/2025) prior to the commencement of the study. The intervention period for the participants was three weeks. All participants were informed about the purpose and procedure of the study, and written informed consent was obtained prior to participation.

Sample size and sampling method

The sample size was calculated using WinPEPI Software version 11.38 (Brixton Health, London) based on the comparison of mean differences between two independent groups. The calculation was performed considering a 95% confidence interval, 80% statistical power, and a significance level (α) of 0.05. An anticipated moderate effect size (Cohen’s d = 0.5) was assumed based on previously published stroke rehabilitation studies evaluating gait parameters following FES and orthotic interventions [15,16]. Based on this effect size, the minimum required sample size was estimated to be 26 participants, with 13 participants allocated to each group.

Participants were recruited using a convenience sampling method from the outpatient and inpatient departments of the study setting based on predefined inclusion and exclusion criteria. Following recruitment, participants were randomly allocated to two intervention groups in a 1:1 ratio using a computer-generated randomization sequence and sealed, opaque envelopes to ensure allocation concealment. Group A received FES combined with AFO, while Group B received FES alone. The sample included individuals diagnosed with ischemic or hemorrhagic stroke presenting with unilateral foot drop.

Inclusion and exclusion criteria

Adults aged 40-60 years with a first-ever ischemic or hemorrhagic stroke of at least six months’ duration, presenting with unilateral foot drop, were included in the study. Participants were required to walk independently for a minimum distance of 10 m, with or without assistive devices, to ensure functional ambulation. Additionally, only participants with a Berg Balance Scale score ≥21 were recruited, indicating mild-to-moderate balance impairment. This criterion was used to include individuals who were ambulatory but still demonstrated residual balance deficits, thereby representing the target population for gait rehabilitation interventions [17]. Participants with severe lower limb contractures, spasticity greater than Grade 3, major cardiopulmonary or musculoskeletal disorders, other neurological conditions, pacemakers or metal implants, unhealed wounds, lower limb deformities, or prior use of FES or AFO were excluded from the study.

Outcome measures

Outcome measures included step length, stride length, cadence, and ground clearance assessed using the Kinovea application (developed by Joan Charmant and Contributors, Kinovea Open Source Project, Bordeaux, France), a reliable video-based motion analysis tool that allows frame-by-frame assessment of gait kinematics with good validity and reliability for clinical and research gait analysis [18]. Video recordings were obtained using a digital camera positioned perpendicular to the sagittal plane at a fixed distance, with a frame rate of 30 frames/second. For spatiotemporal analysis, participants were instructed to walk along a marked 10-m walkway at a self-selected speed.

Cadence was calculated from the video recordings by counting the number of steps taken over the recorded walking duration and converting it into steps per minute using the formula:

$\text{Cadence (steps/min)} = \left(\frac{\text{Number of steps}}{\text{Time in seconds}}\right) \times 60$

Time was determined using frame-by-frame analysis within Kinovea by identifying the initial foot contact of the first and last steps within the defined walkway. Step length and stride length were measured using spatial calibration within the software based on the known walkway distance, while ground clearance was assessed by measuring the vertical displacement of the toe during the swing phase.

Dynamic balance and gait adaptability were assessed using the Dynamic Gait Index (DGI), which demonstrates excellent interrater reliability (intraclass correlation coefficient (ICC) = 0.81-0.98) and intrarater reliability (ICC = 0.89-0.98) [19].

Intervention protocol

Participants in Group A received combined FES and AFO intervention along with conventional gait training, while participants in Group B received FES alone, along with conventional gait training, using identical stimulation parameters. Surface FES was applied to the common peroneal nerve at a frequency of 30-40 Hz and a pulse width of 250-300 µs. Two surface electrodes were used: the active electrode was placed over the motor point of the tibialis anterior muscle, just lateral to the tibial crest in the proximal one-third of the leg, while the reference electrode was positioned distally over the muscle belly of the tibialis anterior or over the common peroneal nerve near the fibular head. The stimulation intensity was adjusted to produce visible and functional ankle dorsiflexion without discomfort and was triggered during the swing phase using a foot switch.

Participants in Group A also used a custom-fitted posterior leaf spring AFO, a dynamic orthosis designed to assist ankle dorsiflexion during the swing phase while allowing controlled plantarflexion during stance. The AFO was worn during gait training sessions and ambulation.

Both groups underwent intervention sessions for 40 minutes/day, five days per week for three weeks. Conventional gait training for both groups included overground walking with therapist feedback, step and cadence training, sit-to-stand activities, weight-shifting exercises, and obstacle-crossing tasks, tailored according to individual ability.

Data collection procedure

Preintervention assessment was performed before the commencement of treatment, and postintervention assessment was conducted after completion of the three-week intervention period. All collected data regarding gait parameters and balance outcomes were compiled using Microsoft Office Excel (Microsoft Corporation, Redmond, WA).

Statistical analysis

Mean and standard deviation (SD) values were calculated for all outcome measures. Statistical analysis was performed using GraphPad InStat software (Dotmatics, Boston, MA). Normality of the data was assessed using the Kolmogorov-Smirnov test. The paired sample t-test was used to compare pre- and postintervention values within groups. The unpaired sample t-test was used to compare pre- and postintervention values between the groups. A p value of <0.05 was considered statistically significant for all analyses.

## Results

A total of 20 men and six women aged 40-60 years, with hemorrhagic or ischemic stroke, volunteered to participate in the study and completed three weeks of the program. Table 1 shows no significant differences between Group A and Group B in baseline characteristics, including age (p = 0.99), sex (p = 0.67), affected side (p = 0.71), DGI (p = 0.18), stride length (p = 1.00), step length (p = 0.06), and cadence (p = 0.52), indicating that both groups were comparable at baseline.

**Table 1:** Baseline demographic data DGI: Dynamic Gait Index; M: male; F: female; FES: functional electrical stimulation; AFO: ankle-foot orthosis

Variable	Group A (FES + AFO) (n = 15)	Group B (FES) (n = 15)	p value
Age distribution (years)			
40-45	15%	15%	0.99
46-50	23%	23%	
51-55	27%	27%	
56-60	35%	35%	
Sex (M/F)	M: 10 (38.4%); F: 3 (11.5%)	M: 9 (34.6%); F: 4 (15.3%)	0.67
Affected side (right/left)	R: 6 (20%); L: 7 (26.9%)	R: 3 (11.5%); L: 10 (33.3%)	0.23
DGI score	13.5 ± 2.14	14.6 ± 2.14	0.18
Stride length (in meters)	0.71 ± 0.02	0.71 ± 0.02	1.00
Step length (in meters)	0.35 ± 0.01	0.34 ± 0.01	0.06
Cadence (steps/minute)	75.5 ± 2.9	76.4 ± 4.15	0.52

Table 2 shows significant improvement in DGI scores in both groups after intervention. Group A improved from 13.53 ± 2.14 to 20.00 ± 1.52 (p < 0.0001), while Group B improved from 14.69 ± 2.14 to 19.00 ± 1.52 (p = 0.001). Greater improvement was observed in Group A.

**Table 2:** Within-group analysis of DGI between Groups A and B The p values were calculated using the paired t-test for within-group comparison ^*^p < 0.01 (very significant); ^**^p < 0.001 (highly significant) SD: standard deviation; DGI: Dynamic Gait Index

Group	Assessment	Mean ± SD	Mean difference	t value	p value
Group A (n = 13)	Pre	13.53 ± 2.14	6.46	8.85	<0.0001^**^
	Post	20.00 ± 1.52			
Group B (n = 13)	Pre	14.69 ± 2.14	4.30	4.74	0.001^*^
	Post	19.00 ± 1.52			

Table 3 presents outcome measures for the paretic limb, showing statistically significant improvements in Kinovea stride length and step length in both groups following intervention (p < 0.0001). Group A showed greater improvement in stride length (0.71-0.88) and step length (0.35-0.46) compared to Group B, which improved from 0.71 to 0.81 and 0.34 to 0.40, respectively.

**Table 3:** Within-group analysis of stride and step length between Groups A and B The p values were calculated using the paired t-test for within-group comparison ^*^p < 0.001 (highly significant) SD: standard deviation

Group	Parameter	Assessment	Mean ± SD	Mean difference	t value	p value
Group A (n = 13)	Stride length (in meters)	Pre	0.71 ± 0.02	0.16	13.06	<0.0001*
		Post	0.88 ± 0.03			
	Step length (in meters)	Pre	0.35 ± 0.01	0.10	14.89	<0.0001*
		Post	0.46 ± 0.02			
Group B (n = 13)	Stride length (in meters)	Pre	0.71 ± 0.02	0.10	9.02	<0.0001^*^
		Post	0.81 ± 0.03			
	Step length (in meters)	Pre	0.34 ± 0.01	0.05	9.46	<0.0001^*^
		Post	0.40 ± 0.01			

**Figure 1:**
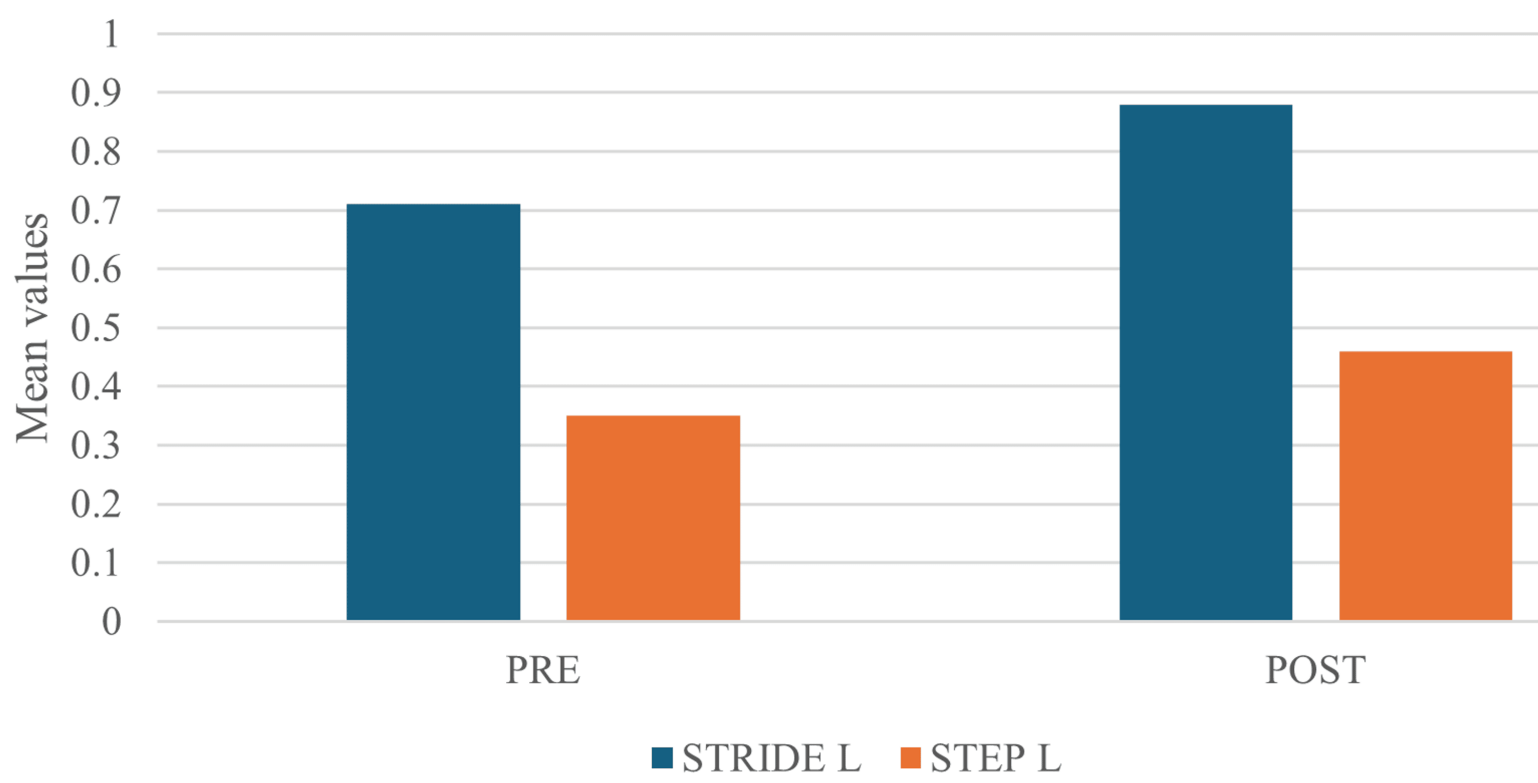
Within-group analysis of stride and step length of Groups A and B

Figure 1 shows improvement in stride length and step length in both Group A and Group B from pre- to postintervention. However, greater improvement was observed in Group A than Group B in both parameters.

Table 4 demonstrates significant improvement in Kinovea cadence in both groups following intervention. Group A showed greater improvement from 75.5 to 90.6 (p < 0.0001), while Group B improved from 76.4 to 81.3 (p = 0.0073).

**Table 4:** Within-group analysis of cadence between Groups A and B The p values were calculated using the paired t-test for within-group comparison ^*^p < 0.01 (very significant); ^**^p < 0.001 (highly significant) SD: standard deviation

Group	Parameter	Assessment	Mean ± SD	Mean difference	t value	p value
Group A (n = 13)	Cadence	Pre	75.5 ± 2.9	15.15	12.81	<0.0001^**^
		Post	90.6 ± 3.0			
Group B (n = 13)	Cadence	Pre	76.4 ± 4.15	0.16	2.93	<0.0073^*^
		Post	81.3 ± 4.27			

**Figure 2:**
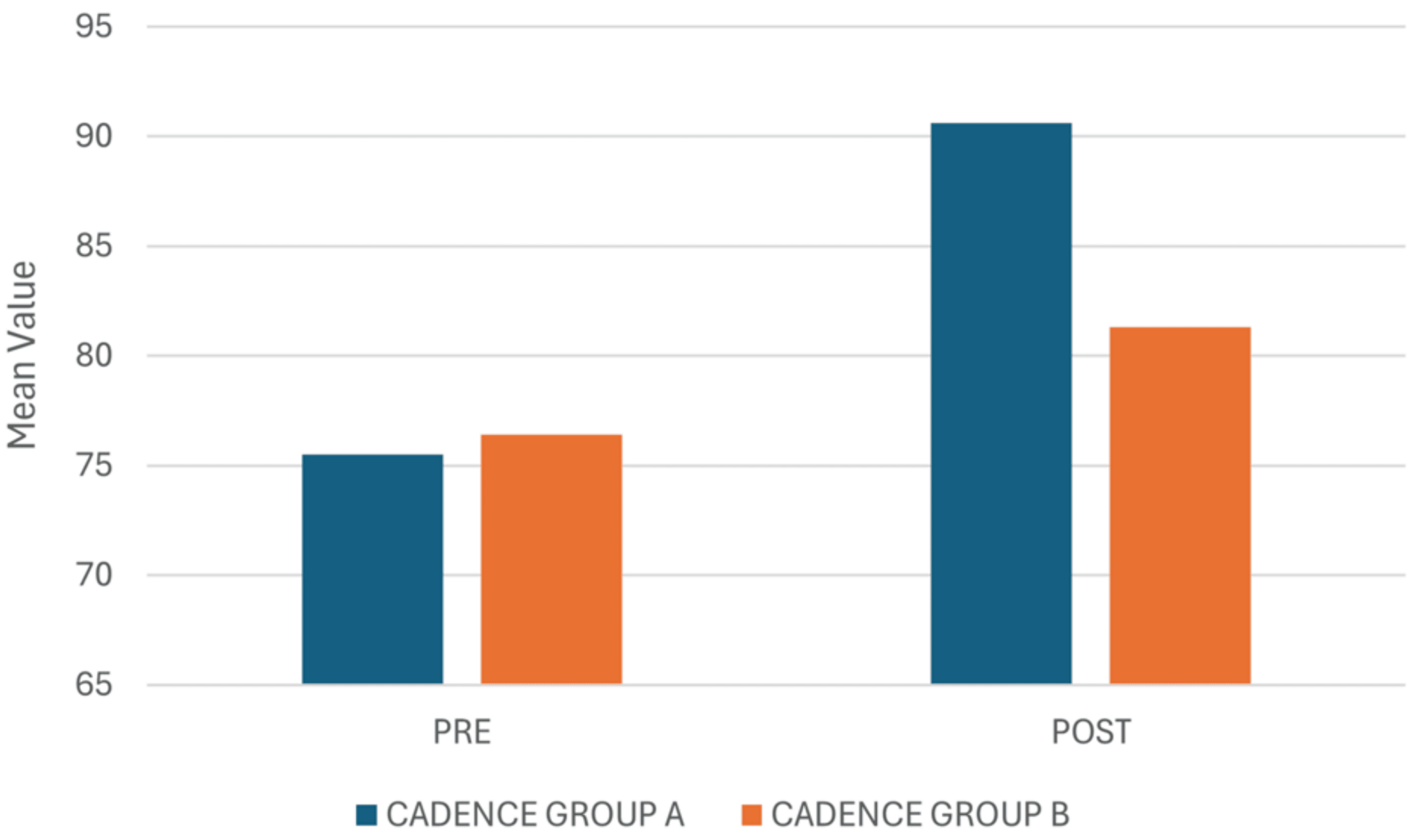
Within-group analysis of cadence of Groups A and B

Figure 2 shows that both Group A and Group B demonstrated an increase in cadence from pre- to postintervention.

Table 5 shows improvement in DGI scores in both groups, with a greater improvement observed in Group A (6.46 ± 1.61) than in Group B (4.30 ± 1.84). The between-group difference was statistically significant (t = 3.170, p = 0.0041), with a large effect size (Cohen’s d = 1.24), indicating a clinically meaningful improvement in Group A.

**Table 5:** Between-group analysis of DGI in Groups A and B The p value was calculated using the unpaired t-test for between-group comparison of DGI scores between Groups A and B ^*^p < 0.01 (very significant) DGI: Dynamic Gait Index; SD: standard deviation

Comparison	Assessment	Mean	SD	Mean difference	t value	p value	Effect size (Cohen’s d)
Group A and Group B DGI	Pre	6.46	1.61	-2.154	3.170	0.0041^*^	1.24
	Post	4.30	1.84				

**Figure 3:**
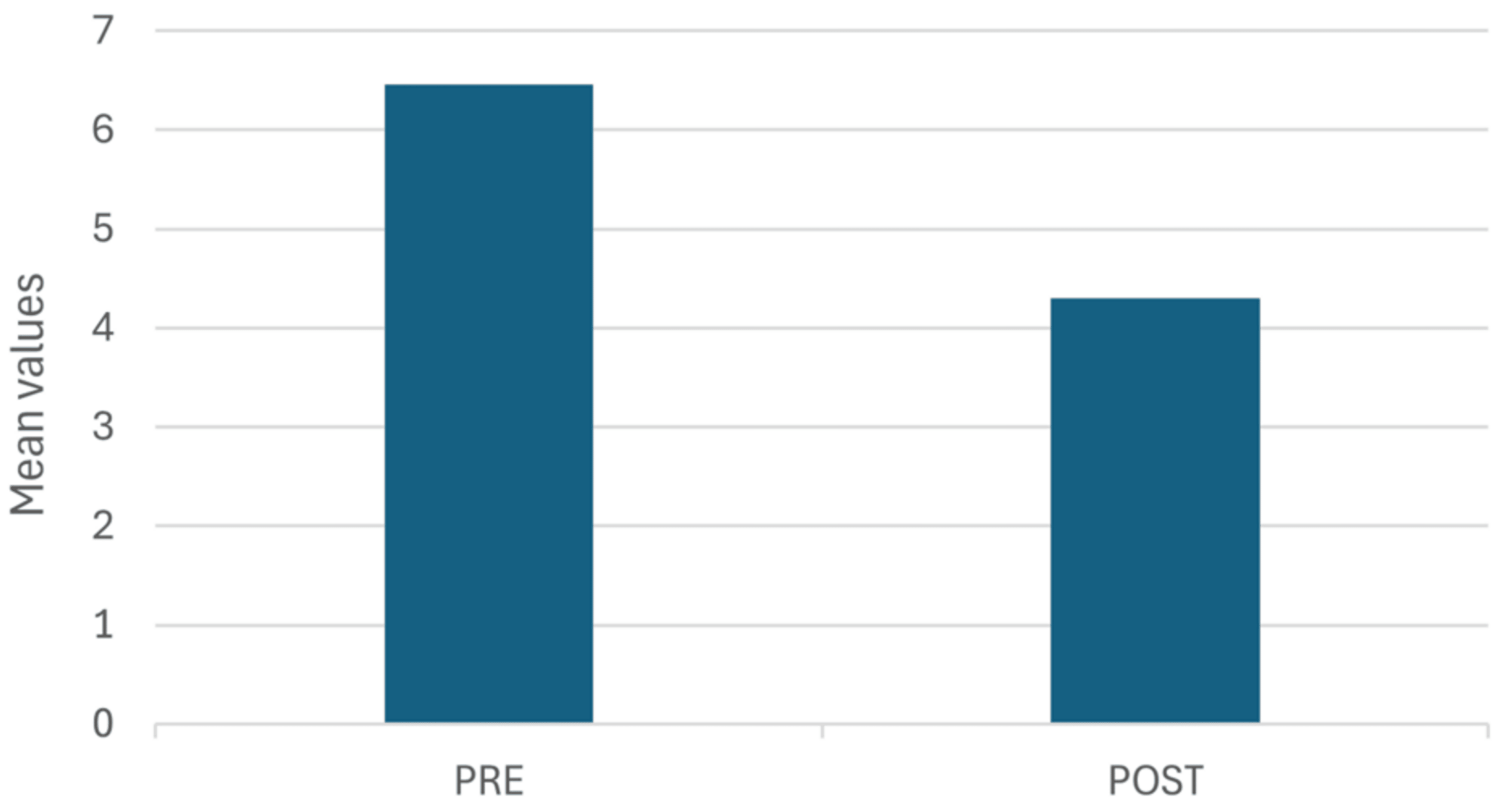
Between-group analysis of DGI DGI: Dynamic Gait Index

Figure 3 shows a reduction in DGI values from pre- to postintervention, indicating a change between the groups, with lower values observed after intervention.

Table 6 demonstrates that stride length and step length were analyzed independently. For stride length, a statistically significant improvement was observed (mean difference = 0.05, t = 6.748, p < 0.0001). Similarly, step length also showed a statistically significant improvement (mean difference = 0.05, t = 7.23, p < 0.0001). Although both variables were statistically significant.

**Table 6:** Between-group analysis of stride and step length The p value was calculated using the unpaired t-test for between-group comparison of stride and step length ^*^p < 0.001 (highly significant) SD: standard deviation

Kinovea analysis		Mean	SD	Mean difference	t value	p value
Stride length	Pre	0.16	0.01	0.05	6.748	<0.0001^*^
	Post	0.10	0.02			
Step length	Pre	0.10	0.01	0.05	7.23	<0.0001^*^
	Post	0.05	0.02			

**Figure 4:**
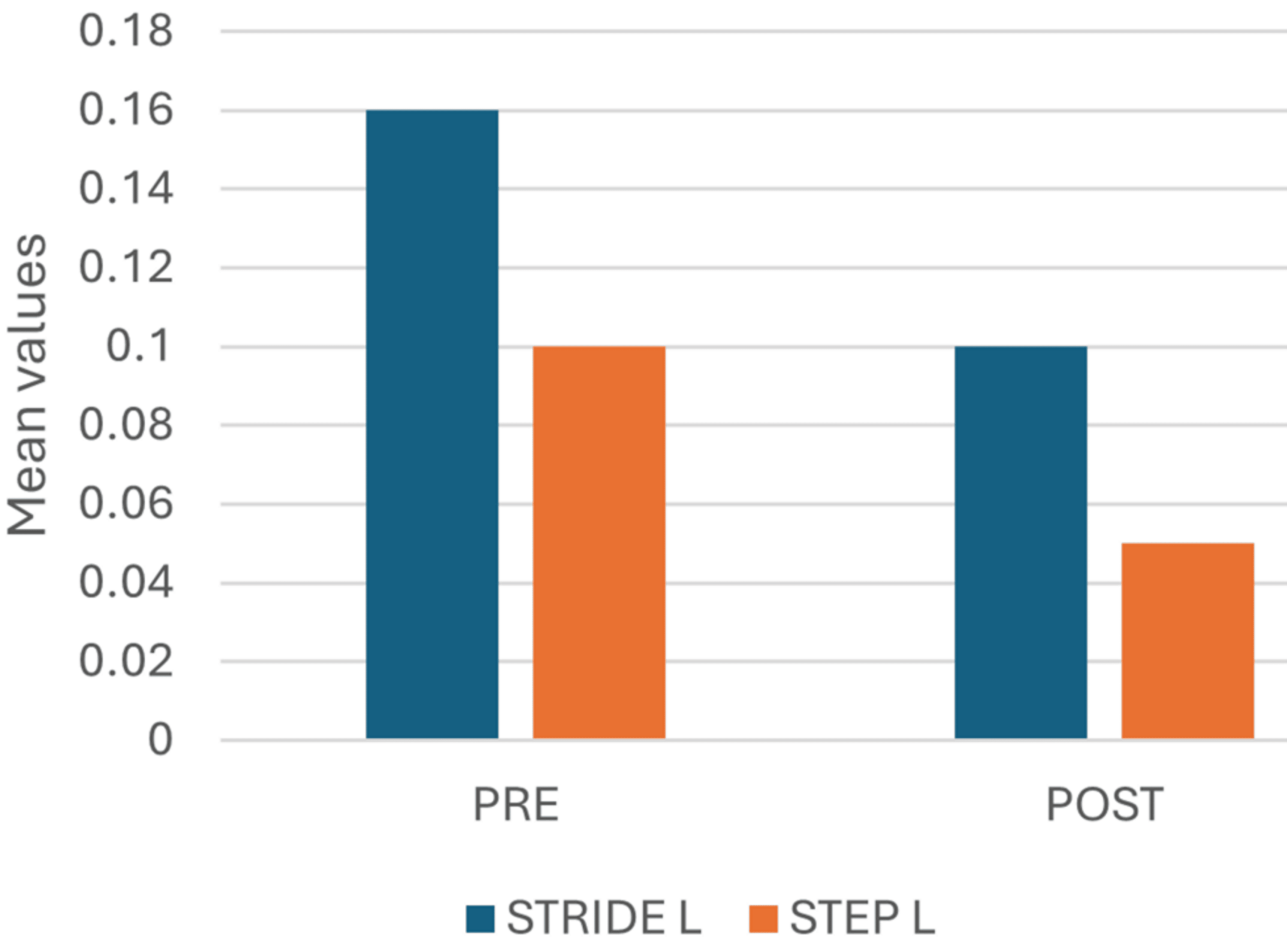
Between-group analysis of stride and step length

Figure 4 shows that both stride length and step length decreased from pre to post between-group comparison, with higher values observed at pre and lower values at post.

Table 7 demonstrates the changes in cadence (steps/minute) following the intervention. A statistically significant improvement was observed, with a mean difference of -10.3 steps/minute (t = 10.90, p < 0.0001). The large effect size (Cohen's d = 4.28) indicates that the observed improvement was not only statistically significant but also clinically meaningful.

**Table 7:** Between-group analysis of cadence The p value was calculated using the unpaired t-test for between-group comparison of cadence ^*^p < 0.05 (statistically significant) SD: standard deviation

Cadence (steps/minute)	Mean ± SD	t value	p value	Cohen's d
Pre	15.5 ± 2.9	10.90	<0.0001^*^	4.28
Post	4.84 ± 1.7			
Mean difference	-10.3			

**Figure 5:**
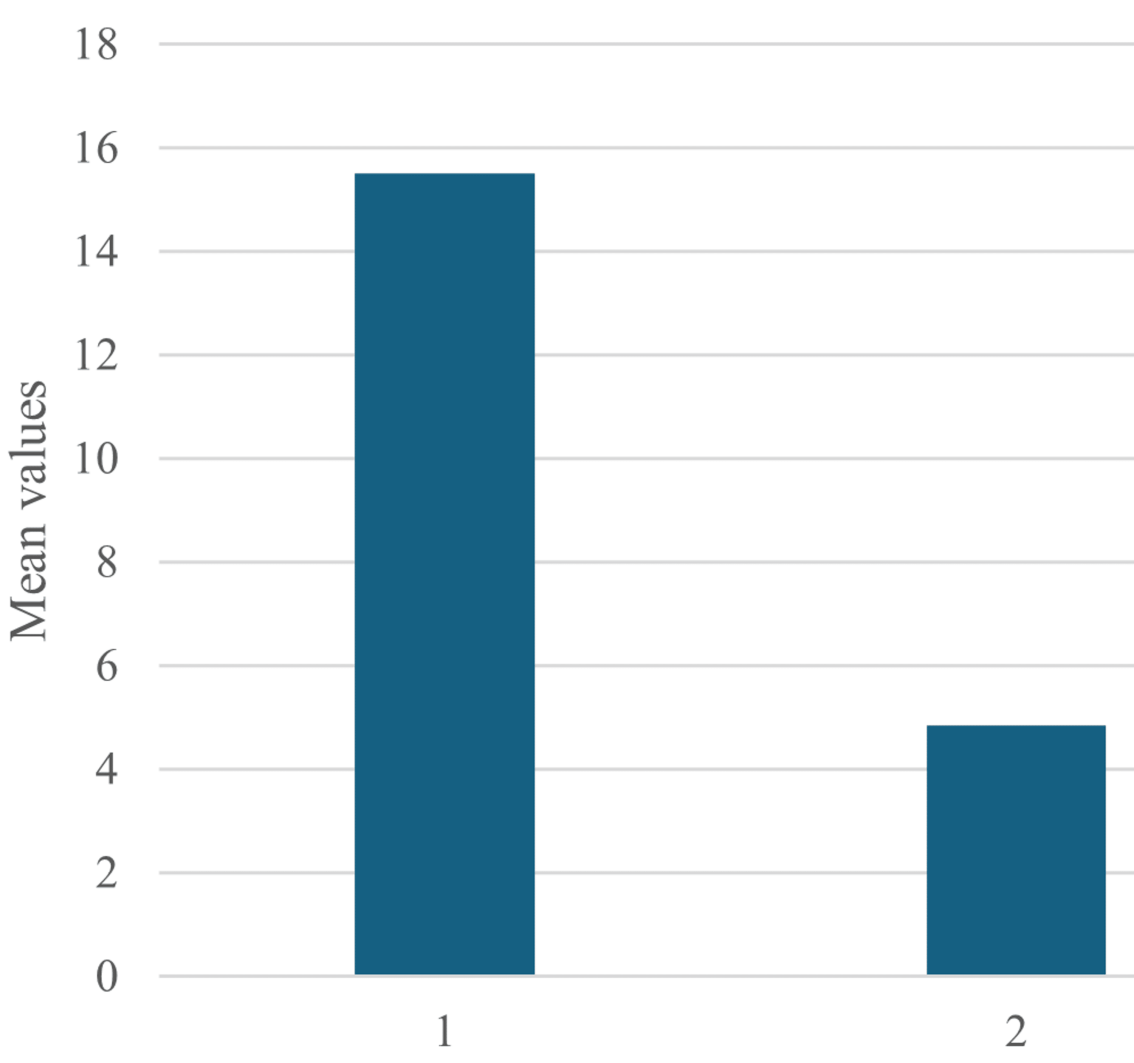
Between-group analysis of cadence 1: Group A; 2: Group B

Figure 5 shows a marked difference in cadence between Group A and Group B, with Group A demonstrating higher values than Group B.

## Discussion

The present study investigated the comparative effects of FES and AFO on gait parameters and ground clearance among stroke survivors with foot drop. Both interventions are established approaches in stroke rehabilitation, yet they function through distinct mechanisms. FES activates dorsiflexor muscles through electrical stimulation of the peroneal nerve to restore active movement, whereas AFO provides passive mechanical stabilization of the ankle joint during gait. The study demonstrated significant improvements in key spatiotemporal parameters, including stride length, step length, cadence, and ground clearance assessed using the Kinovea application, along with dynamic balance measured by the DGI. These findings suggest that the intervention effectively enhanced gait performance and balance, likely through improved neuromuscular control and motor coordination, rather than merely reflecting measurement outcomes. The findings revealed that both FES and AFO produced significant improvements across all measured outcomes, with FES showing comparatively greater enhancement in dynamic gait performance and toe clearance. These results underscore the potential of FES not only as an assistive tool. It also functions as a rehabilitative modality that promotes neuromuscular reeducation and functional recovery [20].

Improvements in stride length and step length observed in both groups indicate better symmetry and efficiency in gait following intervention. In AFO users, the mechanical control provided by the orthosis maintained the ankle in a neutral dorsiflexed position, facilitating consistent step progression. This may have contributed to a reduction in compensatory movements such as hip hiking and circumduction; however, these parameters were not directly assessed in the present study and should be interpreted as a potential mechanism rather than a confirmed finding. In the FES group, repetitive activation of the peroneal nerve promoted active dorsiflexion and improved limb advancement during the swing phase. It is hypothesized that such neuromuscular activation may enhance proprioceptive feedback to the central nervous system, potentially supporting motor relearning and cortical reorganization; however, these mechanisms were not directly evaluated in the present study and should be interpreted cautiously, with support from existing literature. The effectiveness of both AFO and FES in enhancing walking performance has been established; yet, FES is likely to provide better long-term outcomes owing to its potential to activate muscles [21]. Indeed, considerable improvement in stride length after stimulation of peroneal nerves in stroke patients was noted, thus confirming the current results showing that such approaches are efficient in enhancing gait characteristics through the improved dorsiflexion [22].

Cadence, which reflects rhythmic efficiency and step timing during walking, showed notable improvement following both interventions. The use of AFO likely increased confidence and stability during the stance phase, reducing hesitation between steps. FES, on the other hand, enhanced rhythmicity by improving swing initiation and timing through active dorsiflexor control [23]. This improvement in cadence reflects a more coordinated and confident walking pattern. FES improves walking rhythm and step consistency compared to AFO, highlighting its ability to normalize gait patterns by restoring neural control over the ankle musculature and promoting more coordinated and efficient movement during ambulation [24].

Ground clearance during the swing phase is a critical determinant of gait safety and efficiency. The present study found that FES resulted in a greater increase in toe clearance compared to AFO. This improvement can be attributed to the direct activation of the tibialis anterior muscle through timed electrical stimulation of the peroneal nerve, resulting in active dorsiflexion at the appropriate moment during swing. This not only prevents tripping but also reduces the need for compensatory hip and knee flexion movements. AFO also contributed to improved ground clearance by holding the foot in a neutral position; however, because it lacks active muscle engagement, its effect was comparatively limited. These observations align with the results that FES significantly improves minimum toe clearance and gait adaptability in stroke survivors compared with AFO users. This enhanced toe clearance reflects better dynamic control of the ankle and contributes to increased confidence during obstacle negotiation [25,26].

DGI, which evaluates the adaptability and stability of walking under varying conditions, improved significantly in both intervention groups, with greater gains observed in the FES group. This improvement indicates enhanced balance, coordination, and gait performance following active stimulation training. FES may provide augmented sensory input that contributes to improved motor control during walking tasks, although proprioceptive awareness and dynamic gait adaptability were not directly assessed in the present study. In contrast, while AFO supports joint alignment, it may restrict natural ankle motion, thereby potentially limiting adaptability. These findings are consistent with previous studies reporting that FES produces comparable or greater improvements in gait performance and community ambulation compared with AFOs in individuals with poststroke hemiparesis [27].

The mechanisms underlying these improvements can be explained by both biomechanical and neurophysiological factors. Biomechanically, the AFO maintains the ankle in a dorsiflexed position, facilitating heel strike and smoother roll-over during stance, while FES produces active dorsiflexion during the swing phase through stimulation of the peroneal nerve. It is proposed that FES may provide combined afferent and efferent input that could support motor control during gait; however, mechanisms such as cortical reorganization and neuroplastic changes were not directly evaluated in the present study and should be interpreted cautiously. In contrast, AFO primarily offers mechanical support without active muscle engagement, which may limit its effects to the period of device use.

Comparison with existing literature further reinforces these findings. Stein et al. in a multicenter randomized trial reported that both FES and AFO improve gait speed and endurance in chronic stroke, with FES showing higher patient satisfaction and sustained benefits [27]. Similarly, Prenton et al. conducted a systematic review and found that FES and AFO produce comparable effects on gait speed, but that FES is superior in improving dynamic control and voluntary muscle activation [28].

Clinically, these findings have several important implications. Both FES and AFO are effective in improving gait safety and efficiency in stroke survivors with foot drop. AFO remains an excellent option for individuals requiring immediate mechanical stability, especially in those with severe weakness, poor tolerance to electrical stimulation, or limited cognitive capacity. FES, however, offers advantages for individuals with partial motor recovery and preserved peripheral nerve conduction, as it promotes active motor relearning and potentially leads to long-term improvements. Incorporating Kinovea video analysis in the study demonstrated a reliable and accessible method for quantifying gait parameters such as stride length, step length, and cadence.

Despite promising findings, certain limitations must be acknowledged. The sample size was relatively small, limiting the generalizability of results. The duration of intervention was short, potentially insufficient to capture long-term neuroplastic adaptations induced by FES. Kinovea software provided only two-dimensional gait analysis, which may not fully represent the three-dimensional nature of gait kinematics. Participant blinding was not feasible due to the visible differences between devices, potentially introducing bias. Additionally, no electromyographic data were collected to objectively confirm neuromuscular activation during FES. Variations in the design and material of AFOs and differences in electrode placement or stimulation intensity in FES could also influence individual outcomes.

## Conclusions

The study found that both FES and AFO improved gait parameters and ground clearance in stroke survivors. FES demonstrated greater improvements in stride length, cadence, and dynamic balance compared to AFO, while AFO provided immediate mechanical stability. Both interventions contributed to improved walking safety and efficiency. However, the short duration of the study limits conclusions regarding long-term effects.
